# Plasticity to ocean warming is influenced by transgenerational, reproductive, and developmental exposure in a coral reef fish

**DOI:** 10.1111/eva.13337

**Published:** 2022-01-18

**Authors:** Moisés A. Bernal, Timothy Ravasi, Giverny G. Rodgers, Philip L. Munday, Jennifer M. Donelson

**Affiliations:** ^1^ Department of Biological Sciences Auburn University Auburn Alabama USA; ^2^ Marine Climate Change Unit Okinawa Institute of Science and Technology Graduate University Onna‐son, Okinawa Japan; ^3^ ARC Centre of Excellence for Coral Reef Studies James Cook University Townsville QLD Australia; ^4^ College of Science and Engineering James Cook University Townsville QLD Australia

**Keywords:** acclimation, aerobic metabolism, climate change, gene expression, phenotypic plasticity

## Abstract

Global warming is expected to drive some ectothermic species beyond their thermal tolerance in upcoming decades. Phenotypic plasticity, via developmental or transgenerational acclimation, is a critical mechanism for compensation in the face of environmental change. Yet, it remains to be determined if the activation of beneficial phenotypes requires direct exposure throughout development, or if compensation can be obtained just through the experience of previous generations. In this study, we exposed three generations of a tropical damselfish to combinations of current‐day (Control) and projected future (+1.5°C) water temperatures. Acclimation was evaluated with phenotypic (oxygen consumption, hepatosomatic index, physical condition) and molecular (liver gene expression) measurements of third‐generation juveniles. Exposure of grandparents/parents to warm conditions improved the aerobic capacity of fish regardless of thermal conditions experienced afterwards, representing a true transgenerational effect. This coincided with patterns of gene expression related to inflammation and immunity seen in the third generation. Parental effects due to reproductive temperature significantly affected the physical condition and routine metabolic rate (oxygen consumption) of offspring, but had little impact on gene expression of the F3. Developmental temperature of juveniles, and whether they matched conditions during parental reproduction, had the largest influence on the liver transcriptional program. Using a combination of both phenotypic and molecular approaches, this study highlights how the conditions experienced by both previous and current generations can influence plasticity to global warming in upcoming decades.

## 1 INTRODUCTION

The increase in average environmental temperatures as a result of climate change poses a considerable risk to many species, as temperatures may surpass upper thermal limits (Dahlke et al., 2020; Pinsky et al., 2019; Tewksbury et al., 2008). Some species will survive these environmental changes through selection of heat‐resistant genotypes and heritable transmission of thermally suited phenotypes (Munday et al., 2013). However, due to the rapid rate at which environmental change is taking place, it is unlikely that these mechanisms of genetic adaptation will guarantee the persistence of species with longer generation times (Gienapp et al., 2008; Merilä, 2012). Furthermore, theory suggests that the reduction of genetic diversity resulting from a strong selection of heat‐resistant genotypes may be detrimental when species are exposed to fluctuating environments (Orive et al., 2019). The potential limitations of adaptation to rapid temperature changes have re‐sparked the interest in the role phenotypic plasticity can play in the response of organisms to global change (e.g., Sandoval‐Castillo et al., 2020). Furthermore, this classic view of selection of genotypes via adaptation is not exclusive, since adaptive phenotypes can evolve via different genetic mechanisms (Bernatchez, 2016). Hence, understanding when and how phenotypic plasticity occurs in relation to environmental change is essential to predict the adaptive role it may play in response to future climate change (Gibert et al., 2019; Morley et al., 2019; Sandoval‐Castillo et al., 2020).

The thermal sensitivity and plasticity of ectotherms is of particular interest due to the inherent physiological sensitivity and lack of internal temperature regulation. Among marine species, tropical ectotherms tend to be living close to upper thermal limit (Deutsch et al., 2008; Pinsky et al., 2019; Sunday et al., 2011), making them highly relevant to study in the context of global warming. Rising temperatures lead to an increase in cellular metabolic costs (Gillooly et al., 2002; Schulte, 2015), which can lead to an overall decrease of aerobic capacity when the cardiorespiratory system is no longer able to sustain the oxygen requirements of tissues (Farrell, 2016; Pörtner et al., 2017; Pörtner & Knust, 2007). Thus, short‐term warming (days to months) can have a wide array of detrimental consequences for growth rate, body condition, and reproduction (Alfonso et al., 2021; Pankhurst & Munday, 2011; Politis et al., 2017; Rummer & Munday, 2017). Experiments suggest that some of the negative physiological effects experienced with warming can be compensated when lineages experience warming across multiple generations (Donelson et al., 2018; Salinas & Munch, 2012; Shama et al., 2014). Yet, it remains to be determined if the activation of beneficial mechanisms occurs just by exposing previous generations to warm water, or if development of the present generation at elevated temperatures is also required (Donelson & Munday, 2015; Donelson et al., 2018).

Theoretical expectations suggest that the frequency and intensity of environmental variation should favor certain types of adaptive plasticity. Hence, reversible plasticity could be preferred when environmental variation happens within a generation, unpredictable variation between generations should favor developmental plasticity, while transgenerational plasticity would be observed in environments with predictable variation between generations (Herman et al., 2014; Leimar & McNamara, 2015; Reed et al., 2010). Empirically, there is a gap in our understanding on how the interplay between thermal experience from previous and current generations can influence thermal plasticity (Byrne et al., 2020; Donelson et al., 2018). This is due to the limited number of studies on vertebrates investigating multiple generations in sufficient detail to disentangle the relative influence of historical and present thermal conditions on the observed phenotypes (Donelson et al., 2018; Le Roy et al., 2017; Salinas & Munch, 2012). In addition, knowledge gained from model invertebrates may not transfer to other taxa, including fish, given the large differences between life history traits and historical predictability of environmental variation relative to generation time (Chevin et al., 2010; Herman et al., 2014; Reed et al., 2011). Even fewer studies have combined phenotypic and molecular approaches to understand cross‐generational phenotypic plasticity, which is important for understanding the physiological mechanisms that underpin plasticity with the goal of projecting biological effects to future global warming.

The spiny chromis, *Acanthochromis polyacanthus*, is a coral reef fish that has been previously studied in the context of thermal plasticity. Metabolic attributes have been observed to show differing levels of acclimation within and across generations. Resting metabolic rate can be partially restored back to control levels with developmental plasticity (Donelson et al., 2012), while aerobic scope was fully restored within two generations due to enhanced maximum oxygen delivery (Bernal et al., 2018; Donelson et al., 2012). This phenotypic change was accompanied by differential regulation for genes associated with metabolic activity, immunity, and stress response, as well as shifts in methylation of genes for energy homeostasis, mitochondrial activity, and oxygen consumption (Bernal et al., 2018; Ryu et al., 2018; Veilleux et al., 2015). One limitation of these previous studies, however, is associated with the parental care in this species, which has made it challenging to disentangle parental and embryonic offspring from thermal experiences. Consequently, it is still unclear whether the phenotypic plasticity observed in these studies is the result of thermal experience of previous generations, the embryonic conditions of the current generation, or their combination (Torda et al., 2017).

**FIGURE 1:**
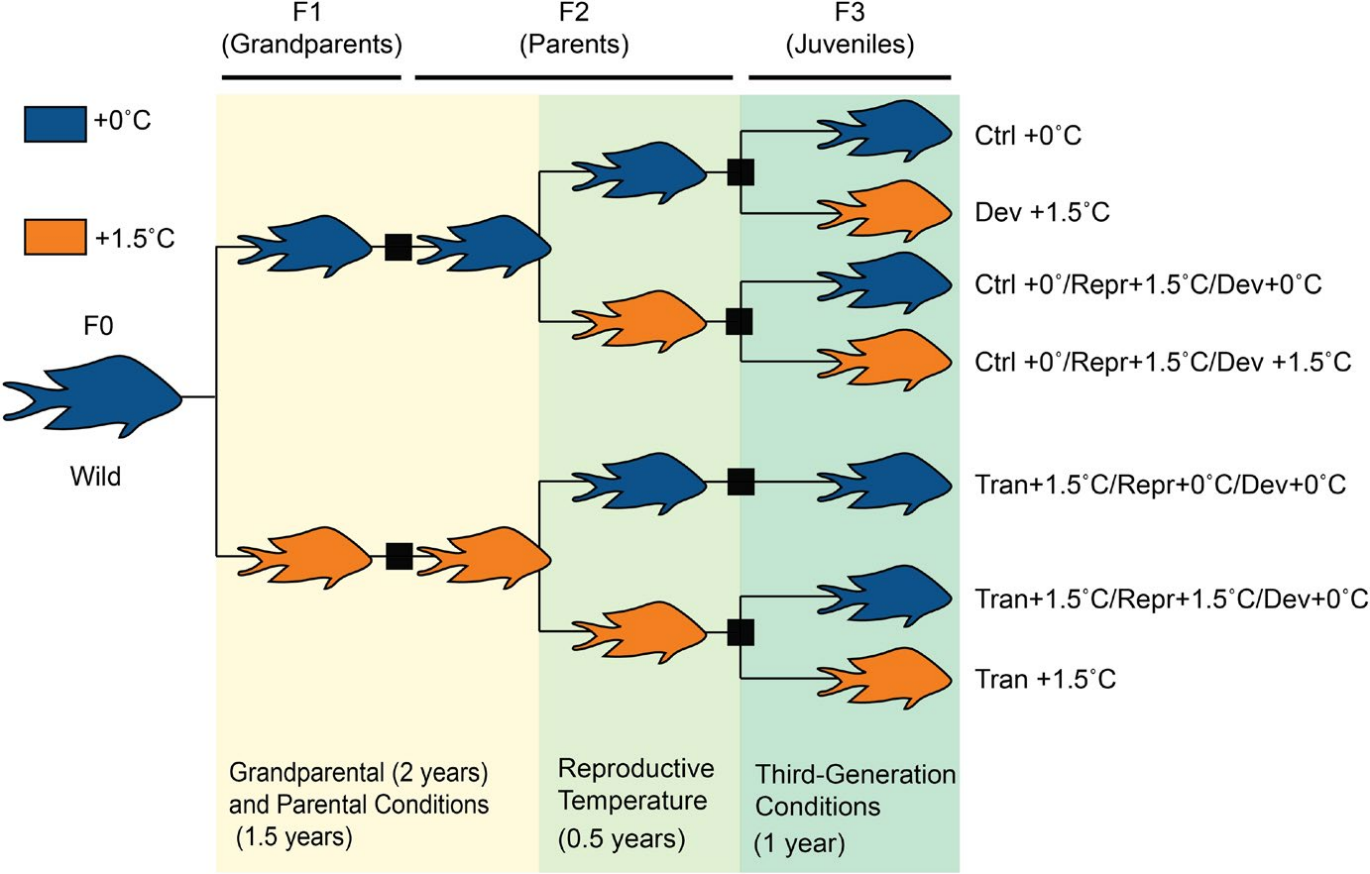
Multigenerational experimental design. The colors represent the temperature treatments: blue for Control +0ºC and orange for Warm +1.5ºC. Background shading represents generational exposure components: yellow = grandparental (2 years) and parental developmental conditions (1.5 years), yellow‐green = reproductive conditions of parents (0.5 years), and green = conditions of F3 juveniles (1 year). Black boxes represent the timing of hatching of each generation in relation to the thermal conditions

While previous studies on *A*. *polyacanthus* focused on the effects of temperature on two generations, this study is based on a three‐generation experiment (Figure 1), which is necessary for disentangling how variable thermal conditions across generations influence plasticity. This experiment included a Control treatment (+0°C) that followed the current average seasonal temperature cycle, and a Warm treatment that matched mid/end‐of‐century projections for ocean warming (+1.5°C; Hobday & Lough, 2011). Phenotypic and molecular traits were analyzed across the different thermal combinations to disentangle the effects of grandparental/parental developmental temperature, parental reproductive temperature, and developmental temperature of F3 juveniles. The goals of this study were to: (1) determine which phenotypic and molecular responses to warming are due to transgenerational effects (i.e., exposure of grandparents/parents); (2) evaluate if transgenerational effects differ depending on parental reproduction and embryonic conditions of the offspring; (3) assess the influence of parental effects due to reproductive thermal conditions; and (4) explore how thermal experience of previous generations interacts with the developmental conditions of the third generation. The results from this long‐term study represent a step forward in understanding the limitations of transgenerational plasticity, which is essential to elucidate the response of organisms to climate change over coming decades. This experiment is also relevant from an ecological perspective, as it provides a time frame over which we can expect phenotypic changes to take place in the presence of ocean warming.

## 2 MATERIALS AND METHODS

### 2.1 Experimental design

The spiny chromis, *A*. *polyacanthus*, is a coral reef damselfish that forms monogamous breeding pairs and possesses direct development (i.e., no pelagic larval phase; Pankhurst et al., 1999; Robertson, 1973). This damselfish inhabits coral reefs of the Indo‐Australian archipelago in the Western Pacific (15°N–26°S and 116°E–169°E) and is common on the Great Barrier Reef (Randall et al., 1997).

Eight adult breeding pairs of *A*. *polyacanthus* were collected in August of 2007, from the Palm Island region (18°37′S, 146°30′E) in the central Great Barrier Reef, Queensland, Australia. These wild‐caught fish were maintained in aquaria at James Cook University under current‐day summer water temperatures (28.5°C). Breeding pairs produced F1 offspring that were divided into two seasonally cycling temperature treatments shortly after hatching for the present study (Figure 1; for further details, see Donelson et al., 2012; Donelson et al., 2014). The current‐day Control treatment (+0°C) followed the average seasonal temperature cycle experienced by fish at the collection site, where the average winter and summer temperatures are 23.2°C and 28.5°C, respectively (temperature records at 6–8 m are available at http://data.aims.gov.au/). The elevated temperature treatment matched projections for ocean warming by the end of the century under moderate (+1.5°C) emissions scenarios (Hobday & Lough, 2011; IPCC, 2014), seasonally cycling as in the Control. F1 sibling individuals developed in these treatments until 2 years of age, at which time they were mature and reproductively active. During the 1st year of life, fish were maintained in sibling tanks (42–60 L, n = 3 per treatment) with density reduced from 6 to 2 individuals at the time of pairing. All F1 fish were paired with another fish from an unrelated family, prior to 2 years of age (for further details, see Donelson et al., 2012, 2014). The genealogy of each fish was tracked using a combination of colored elastomer tags.

During the Austral summer of 2009–2010, nonsibling F1 pairs produced F2 juveniles, which hatched at the same temperature as the parents and continued to be reared in these conditions until 1.5 years of age, at which time fish were sexually mature. At 1.5 years (during winter 2011), fish were elastomer tagged, paired, and then randomly split between two of the reproductive temperature treatments (+0 or +1.5°C; Figure 1). Given the reproductive traits of *A*. *polycanthus*, for each generation the offspring experienced the initial stage of development in the same temperature conditions as their parents. In the particular case of F3 offspring, broods were split between the two temperature treatments (+0 or +1.5°C) in all thermal treatment lines at the time of hatching. The only exception was the Transgenerational +1.5°C/reproduction +0°C treatment, which was only reared at +0°C conditions (Figure 1). This treatment was omitted due to the lack of available space in the aquarium facility, and its absence in the present analyses is unrelated to mortality or hatching success of F3 individuals. Development of F3 fish in thermal conditions continued until the austral summer of 2013 (i.e., 1 year). Throughout this time, F3 fish were maintained in sibling groups of 2–6 individuals (n = 2–3 tanks per pair/treatment).

One potential outcome when running multigenerational experiments in the laboratory is selection of breeding lines that perform better at warm temperature. However, we did not observe evidence for this in previous generations (Donelson et al., 2012) as reproduction of *A*. *polyacanthus* is not affected by +1.5°C, which minimizes the potential of selecting heat‐resistant genotypes (Donelson et al., 2016).

Finally, this research was conducted under the James Cook University animal ethics A1233, A1415, and A1547. The original collection of wild fish in 2007 was completed under Great Barrier Reef Marine Park and Queensland Fisheries Permits G06/20234.1 and 103256, respectively.

### 2.2 Phenotypic traits

Sample sizes for the phenotypic and molecular measurements for each thermal treatment of the F3 are available in Table 1, as well as Table S1 (samples divided by sex) and Table S2 (samples divided by family). At the end of summer in 2013, when F3 fish were between 1 year and 1 year 4 months of age, aerobic metabolic traits and physical condition were explored between the seven treatment lines (Figure 1). For all of the F3 treatment groups, aerobic metabolic traits were tested at their respective summer treatment temperatures. Specifically, the summer temperatures were 28.5°C in the +0°C treatment (Control) and 30.0°C in the +1.5°C treatment (Warm). Routine oxygen consumption (MO_2Routine_) and maximum oxygen consumption (MO_2Max_) were measured as a proxy for metabolic rate. Fishes were starved for 12–24 hr prior to testing to eliminate any effects caused by digestion. For MO_2Routine_, fish were placed in 0.59‐ or 1.19‐L transparent plastic respirometers (matched to fish mass) and allowed to acclimate for 1 hr with constant water flow prior to testing. Placement of fish into chambers involved gently corralling the fish and allowing them to swim into the chamber, thus, there was no stress caused by handling or netting. This short habituation time has been found to be sufficient to achieve routine measurements in this species with this handling method (Donelson et al., 2012; Rodgers et al., 2019). Following habituation, the chamber was sealed and oxygen concentrations were measured for 30–40 min to determine MO_2Routine_ (measured with PreSens Fibox 3 noninvasive optical setup). Static measures produce consistent and reliable measures of MO_2Routine_ for this species, likely due to natural constant pectoral fin movement (Donelson et al., 2012; Rodgers et al., 2019).

**TABLE 1:** Sample sizes for the phenotypic (oxygen consumption, hepatosomatic index ‐ HSI, and body condition) and molecular (liver gene expression) measurements for each of the temperature treatments of the F3 fish

Treatment	Oxygen consumption	HSI and body condition	Gene expression
Control +0°C	21	8	7
Developmental +1.5°C	12	9	8
Control +0°C /reproduction +1.5°C/development +0°C	9	9	8
Control +0°C /reproduction +1.5°C/development +1.5°C	25	6	6
Transgenerational +1.5°C/reproduction +1.5°C/developmental +0°C	16	6	6
Transgenerational +1.5°C/reproduction +0°C/ development +0°C	20	11	7
Transgenerational +1.5°C	17	7	5

All chambers were washed between trials to remove buildup of biological material. Background respiration was found to be negligible at all testing temperatures, and this was consistent with a previous work showing it takes close to 6 hr for background respiration to have a significant effect (Rodgers et al., 2016). Measurements of MO_2Max_ were obtained for the same fish, at least 3 hr after MO_2Routine_ measurements, by moving each individual to a circular swim chamber (2.675 L) set to the maximum aerobic swimming speed of the fish (Donelson & Munday, 2012; Seebacher et al., 2013). Each fish was swum for 10 min, and the steepest 5 min of the trial was used for analysis (PyroScience FireSting). Both MO_2Routine_ and MO_2Max_ were calculated in mgO_2_kg^−1^h^−1^. The net aerobic scope of each individual was obtained by subtracting the MO_2Routine_ from the MO_2Max_. For all trials, the respirometer was submerged in a temperature‐controlled aquarium to maintain a stable temperature.

Posteuthanasia, standard length (SL) in mm, total wet weight to nearest 0.01 g, and liver weight to nearest 0.001 g were measured. Body condition was calculated as the linear relationship between SL and W, that is, weight for a given SL. Hepatosomatic index (HSI), a measure of the relative liver size across treatments, was calculated by dividing liver weight by the total weight.

To explore the influence of generational exposure on phenotypic trait response, linear mixed effects models (LMER) were run with R (v 3.4.4; R Core Team, 2013). The independent factors in the model were the grandparent/parent developmental temperature, parent reproductive temperature, and offspring developmental temperature (all fixed). The random factor of parent ID was also included in the model, as a way of controlling for the interrelatedness of sibling F3 individuals. For the metabolic traits MO_2Routine_, MO_2Max_, and net aerobic scope, fish mass (i.e., wet weight) was used as a covariate in the model, as well as both mass and metabolic rate being log transformed to adhere to the statistical assumptions. As the design was not fully orthogonal, exploring the effect of grandparent/parent developmental temperature and parent reproductive temperature was completed with the available comparisons. Here, only F3 fish that developed in +0°C conditions were used to explore the relationship between grandparent/parent developmental temperature and reproductive temperature of parents. This allowed an exploration of the effects of grandparental/parental generation exposure without the influence of the developmental temperature of the third generation. The effect of the thermal experience of previous generations and current developmental temperature was explored with all data except F3 offspring from the Transgenerational +1.5°C/reproduction +0°C, as these fish were not reared at both +0°C and +1.5°C in the F3 generation. To determine the differences due to the thermal experience of previous generations, planned comparisons were run between Control +0°C and Control +0°C/reproduction +1.5°C, as well as Control +0°C/reproduction +1.5°C and Transgenerational +1.5°C to test the presence of an interaction between historical thermal experience and F3 development conditions. Differences between traits dependent on F3 developmental temperatures (+0 or +1.5°C) were also evaluated.

### 2.3 Analysis of gene expression

The molecular responses were evaluated through liver gene expression, as previous studies indicate this organ is well suited for evaluating the effects of temperature in fishes (Smith et al., 2013). Experimental fish of 1 year of age were euthanized by pithing, and the liver of each individual (average weight was 0.13 g) was homogenized and frozen in liquid nitrogen. RNA was extracted from homogenate using the RNeasy Mini kit (Qiagen) following manufacturer's instructions. RNA‐Seq libraries were prepared with the Illumina TruSeq RNA and were sequenced with Illumina Hiseq4000 (150 bp PE), at Macrogen Inc, Korea. Only extractions between 0.8 µg and 1.5 µg of total RNA and a minimum RNA integrity number 7.2 were used for library preparation and sequencing. To avoid sequencing biases, 11 randomly selected samples were sequenced per lane, so that no more than two samples of one treatment were in the same lane. The sample numbers per treatment for the analyses of gene expression are available in Table 1.

Adaptor trimming and removal of low‐quality reads (Q‐score <30) was done using the program Trimmomatic (Bolger et al., 2014). Samples were mapped to the genome of *A*. *polyacanthus* (ENSEMBL ID ASM210954v1; GenBank Assembly ID GCA_002109545.1) using HISAT2 (Kim et al., 2015). RNA‐Seq read counts were summarized to transcripts using featureCounts of the Subread Package (Liao et al., 2014), and the measure of mapping confidence to the reference genome (i.e., Mapping Quality Score) was set to 20. Only RNA‐Seq reads between 50 and 150 bp and with both pairs successfully aligned to reference transcripts were considered for the analysis. Gene counts of expression for 34,194 transcripts were exported to a text file of read counts for downstream analyses. Out of these, 18,716 (55%) had annotation to gene level in the reference genome, while 19,080 (56%) were annotated to Gene Ontology (GO) category. After removal of adaptors and low‐quality reads, samples had on average 34,480,533.4 read counts (SD ± 5,475,895.09) before normalization.

The statistical analyses for gene expression were done with DeSeq2 (Love et al., 2014) in R (v 3.4.4; R Core Team). Sequencing outliers can result from differences in sequencing efforts of RNA libraries, and these could lead to higher or lower number of reads for certain individuals that are unrelated to the temperature treatments. Since these outliers can bias the interpretation of the results, they were removed based on a heatmap of sample distance based on the raw read counts. This resulted in the removal of one Control sample and one Transgenerational +1.5°C/reproduction +1.5°C/development +0°C.

For the analyses of gene expression, a likelihood ratio test (LRT) was conducted to evaluate the effects of warming during the three specific stages (grandparent/parental development, parental reproduction, and F3 development), separating the samples based on temperature profiles (Control +0°C or Warm +1.5°C; Figure 1). The LRT examines if there is a significant difference in likelihood between a full model (effect of all three stages), and a reduced model that excludes one of the conditions (e.g., parental reproduction). To visualize the patterns of differentiation, a Principal Coordinate Analysis (PCoA) was completed using the variance stabilized counts of the differentially expressed genes (DEGs) (*padj*. < 0.01) of the LRT analysis of F3 juvenile condition (given that this was the largest contributor to DEGs). A second approach consisted of performing pairwise comparisons between all seven treatments, using the “Contrast” function of DESeq2. This function tests if deviations in the dispersion of the read counts between two treatments are significantly different from zero, using an adjusted p‐value (*padj* < 0.01). Cook's distances were used to assess the presence of outlier read counts, and at least six samples had to be present in a treatment for the outlier to be replaced by the average number of counts. In treatments with <6 samples, the gene with outlier reads was eliminated from the analysis.

For all pairwise comparisons, we examined if there was enrichment of Gene Ontology (GO) categories using the Mann–Whitney *U* test, with a Benjamini–Hochberg correction (GO‐MWU; available at: https://github.com/z0on/GO_MWU; Wright et al., 2015). This analysis is a test of ranks to determine if genes that belong to a particular GO category are overrepresented at the top (upregulated) or at the bottom (downregulated) of the distribution. The analysis was based on the Log2Fold Change of all genes resulting from the pairwise comparisons in DeSeq2, which has been suggested as a well‐suited measure for the test of ranks (Wright et al., 2015). The analyses were done independently for each of the GO subcategories such as biological process (BP), cellular component (CC), and molecular function (MF). The GO categories were considered enriched if they included more than five terms and passed a 10% false discovery rate (FDR) after the Benjamini–Hochberg correction. The GO terms were collapsed into one if they had more than 75% of similarity in their composition. The results of this analysis are presented in Data S2–S11, and dendrograms of the significant GO categories that were up‐ and downregulated in Figures S2‐S4.

To assess correlation in the patterns of expression of the analyzed genes, and determine which of the gene modules were associated with the experimental conditions, we performed an unsigned Weighted Gene Correlation Network Analysis (WGCNA; Langfelder & Horvath, 2008) in R. For this analysis, transcripts with average counts <10 were removed from the dataset, resulting in a subset of 13,152 genes, which were normalized using the “variance Stabilizing Transformation” function of DESeq2. For the gene module selection, the unsigned adjacency analysis had a soft threshold of 6, and the dendrogram of genes was constructed following average hierarchical clustering. The size of the module was set between 30 and 5000, and different modules were merged if they had 80% similarity in their expression profiles. The association between the resulting gene modules and sample traits was determined with a Pearson correlation, and a heatmap was plotted to display the strength and corrected p‐value of the correlation. The traits considered for the correlations were: grandparental conditions, temperature at time of reproduction, developmental conditions of juveniles, whether the juveniles experienced a thermal mismatch with respect to reproductive parental temperature, and sex of the individual. An analysis of GO enrichment was performed to determine if the genes contained in the modules that showed significant correlation (*p* < 0.05) with the experimental traits had overrepresented GO categories. This analysis was performed following the Fisher's test of enrichment, where the genes contained in a specific module were compared to the complete list of genes in all modules using the program GO‐MWU (Wright et al., 2015), running each of the three subcategories (BP, CC, and MF) independently.

## 3 RESULTS

### 3.1 Transgenerational carry‐over effects

**FIGURE 2:**
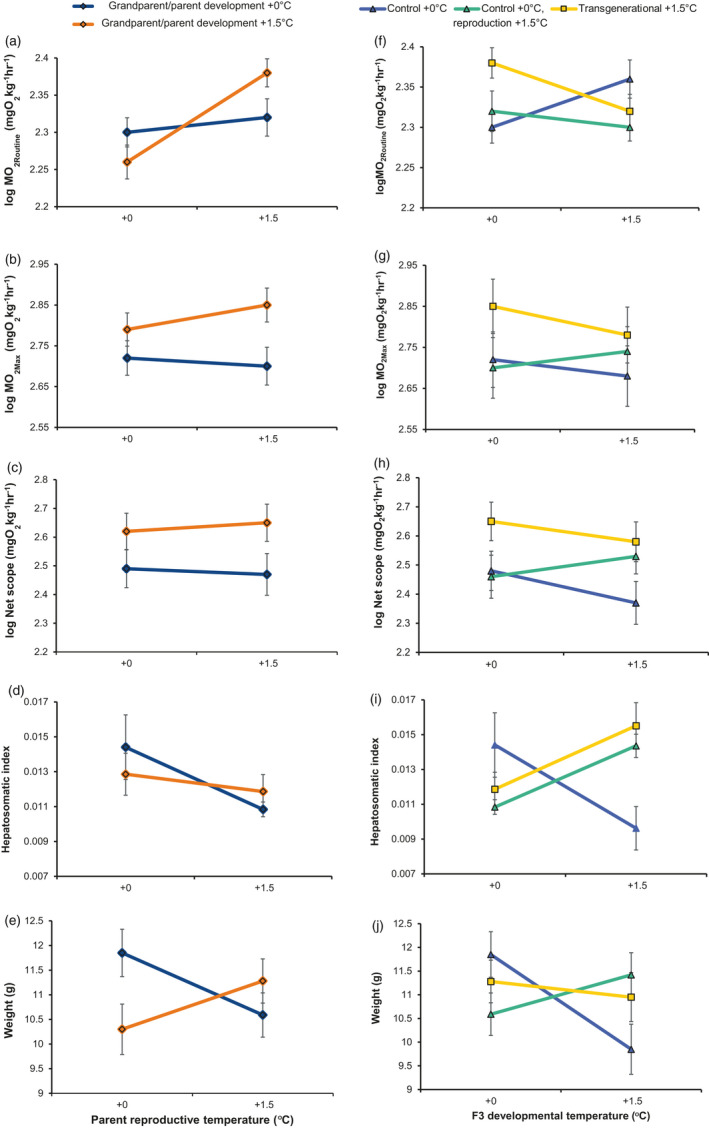
Phenotypic effects of thermal conditions over generations. Left column displays the effect of temperature during grandparent and parent developmental conditions (F1–F2) and parent reproductive conditions on F3 MO_2Routine_ (a), MO_2Max_ (b), net aerobic scope (c), hepatosomatic index (HSI) (d), and body condition: weight for a given standard length (SL) (e), when F3 generation develops at +0°C control conditions (estimated marginal means ±SE). The right column shows the effect of parental reproductive conditions and F3 developmental conditions on phenotypic traits (f–g) for fish from Control +0°C, Control +0°C/reproduction at +1.5°C, and Transgenerational +1.5°C lines

Developmental temperatures of grandparents and parents led to significant differences in maximum oxygen consumption (MO_2Max_: t = −2.520, *p* = 0.020) and aerobic scope (AS: t = −2.299, *p* = 0.031) in the third generation, when individuals developed at Control +0°C (Figure 2B,C; Tables S3–S4). F3 fish from grandparents/parents that developed at +1.5°C had higher MO_2Max_, regardless of the thermal conditions applied during parental reproduction and F3 embryogenesis. The higher MO_2Max_ in F3 fish from the Transgenerational +1.5°C lineage was most pronounced when development occurred at Control +0°C (Figure 2G). A decline in MO_2Max_ in Transgenerational +1.5°C offspring with development at +1.5°C resulted in a significant difference compared with the Control +0°C/reproduction +1.5°C lineage in their interaction with F3 developmental conditions (t = −2.059, *p* = 0.042; Table S3). This transgenerational result was corroborated by the lack of a significant interaction between parental reproductive conditions and F3 developmental conditions for MO_2Max_ between the Control +0°C and the Control +0°C/reproduction +1.5°C lines (Figure 2G; t = 1.478, *p* = 0.143).

Historical thermal exposure also resulted in DEGs between lineages. The LRT with DESeq2 showed differential expression of two genes associated with inflammatory response and immunity, based on the developmental temperatures of grandparents/parents, regardless of parental reproduction and F3 developmental temperature (Data S1). The transgenerational influence on expression was also investigated via pairwise comparisons of F3 fish whose only difference was the developmental temperature of grandparents/parents. Thus, groups with grandparents/parents at Control +0°C compared to parents/grandparents at Transgenerational +1.5°C showed 69 DEGs (parental reproduction and F3 development at +0°C) and 13 DEGs (parental reproduction and F3 development at +1.5°C). After the pairwise comparisons, we assessed the enrichment of Gene Ontology (GO) categories, and these analyses showed the activation of circadian rhythm regulation, immune response, lipid metabolism, DNA replication, GTPase activity, and cofactor binding for treatments with Control +0°C grandparents/parents (Data S2). Meanwhile, groups with grandparents/parents that developed at +1.5°C showed activation for categories associated with catabolism, metabolism of nucleic acids, oxidoreductase activity, signal transduction, and movement of cellular components (Data S3).

### 3.2 Parental reproductive effects

The effect of temperature during parental reproduction and grandparent/parent development resulted in significant interactions for routine oxygen consumption (MO_2Routine_; Figure 2A; t = 2.816, *p* = 0.008; Table S5) and body condition (Figure 2E; t = 2.397, *p* = 0.028; Table S6) for F3 individuals that developed at +0°C. Specifically, the reproductive temperature of the parents in the +1.5°C lineage was associated with reduced MO_2Routine_ when parents reproduced at +0°C, and elevated MO_2Routine_ when parents reproduced at +1.5°C (Figure 2A). In contrast, reproductive temperatures had no effect on the MO_2Routine_ when grandparent/parents experienced +0°C conditions. No difference was observed in body condition based on grandparent/parent thermal conditions when reproduction occurred at +1.5°C. However, offspring from Control +0°C grandparent/parents showed higher body condition than +1.5°C grandparents/parents when reproduction occurred at +0°C.

When considering F3 developmental conditions, we observed a significant influence of parental reproductive temperature (i.e., parental effect) on traits associated with physical condition (Figure 2I and J). There were effects on both hepatosomatic index (HSI) and body condition when F3 fish from the +0°C lineage (i.e., grandparents/parents and reproduction at +0°C) developed at +1.5°C. This resulted in offspring of the grandparent/parent Control +0°C line having poorer physical condition with development at +1.5°C (i.e., experiencing warm temperatures for the first time after hatching), compared to offspring from the Control +0°C/reproduction +1.5°C parents (body condition: t = 3.995, *p* < 0.001; HSI: t = 3.927, *p* = 0.003). Meanwhile, offspring from the Control +0°C/reproduction +1.5°C and the Transgenerational +1.5°C lineage had a similar body condition, regardless of the F3 developmental temperature (Figure 2I and J; Tables S6–7). Offspring of the grandparent/parent Control +0°C and Control +0°C/reproduction +1.5°C groups also differed in AS (Figure 2H; t = 2.058, *p* = 0.042), with reproduction at +1.5°C resulting in higher AS when F3 fish developed at +1.5°C. However, this effect was largely driven by reduced MO_2Routine_ (t = −1.858, *p* = 0.066) rather than an increase in MO_2Max_ at +1.5°C (t = 1.478, *p* = 0.143).

The LRT of liver gene expression showed that the combined effect of grandparent/parent development and reproductive temperatures of parents resulted in 18 DEGs, associated with immune response, inflammation, cellular transport, and cellular stress (Data S1). The effect of changing reproductive temperature was evaluated through pairwise contrasts between fish with the same grandparent/parent and F3 developmental conditions, but differing parental reproductive conditions. In line with the phenotypic results described above, there were 74 DEGs between F3 fish whose parents reproduced at different temperatures (grandparent/parent and F3 development at Control +0°C). This resulted in the activation of GO terms associated with electron transport activity, oxidoreduction, mitochondrial activity, myosin complex, toxin metabolism, cell growth, and circadian regulation of expression for individuals whose parents reproduced at +1.5°C (Data S4). The opposite comparison with F3 development at +1.5°C was not possible, as a treatment was absent from our study due to lack of space in the experimental facilities (Figure 1).

### 3.3 Effects on third‐generation juveniles

**FIGURE 3:**
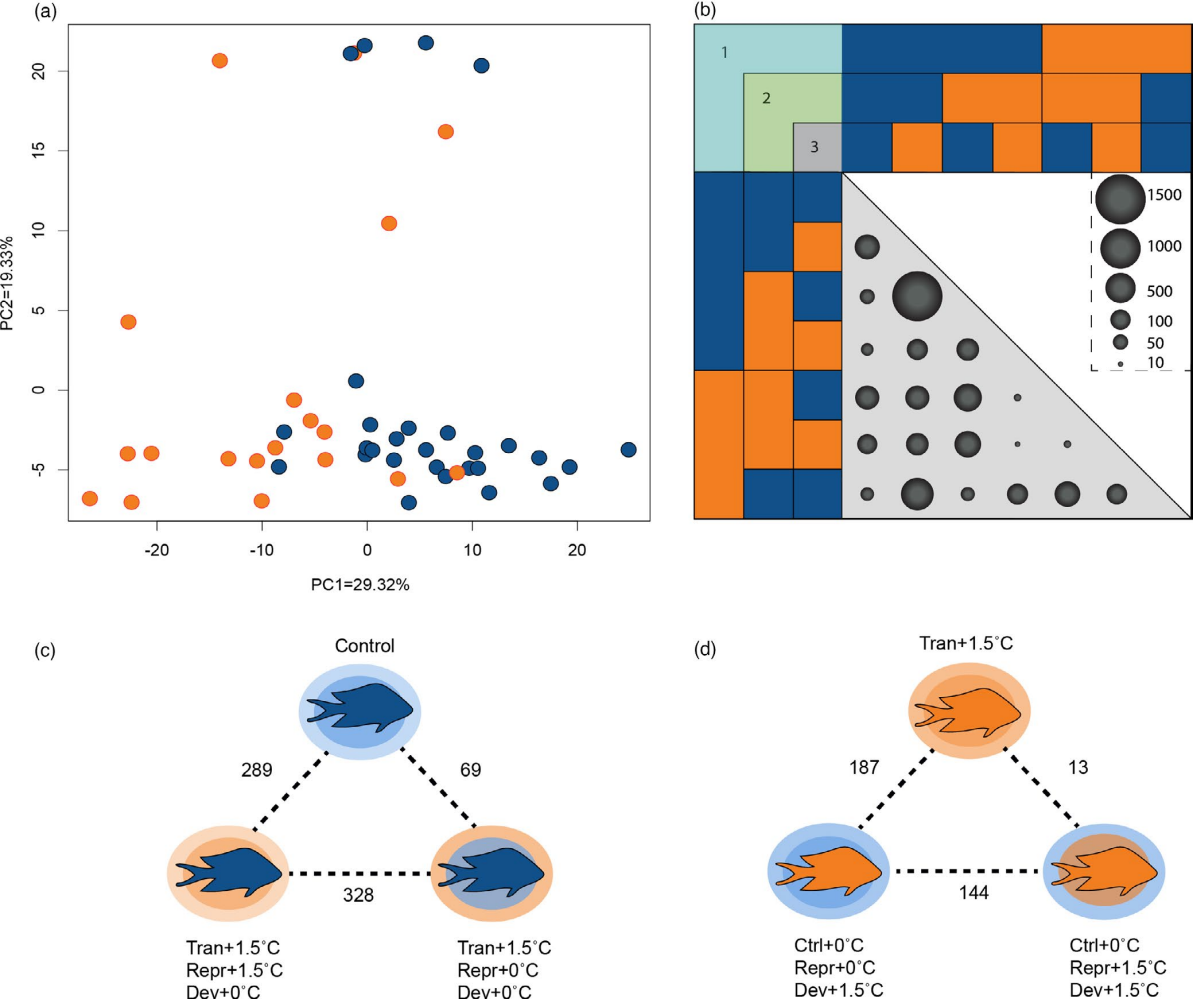
Differential gene expression for F3 fish. (a) Principal Coordinate Analysis (PCoA) of F3 juveniles based on 459 differentially expressed genes (DEGs) between Control (blue) and +1.5°C (orange) developmental conditions, excluding DEGs from grandparental/parental and reproductive temperatures. Developmental temperature of F3 was the biggest axis of differentiation for RNA‐Seq analyses (PC1 = 29.32%), while the second axis is driven by individuals with mismatch between parental reproduction and development (PC = 19.33%; all treatments depicted in Figure S1). (b) DEGs for the pairwise comparisons of F3 juveniles. Numbers on the top‐left corner represent the conditions at different stages: grandparental/parental development (1), parental reproduction (2), and F3 development (3). The size of the circles is proportional to the number of genes significantly differentiated between comparisons (*p* < 0.01). (c) DEGs between Control +0°C (top), Transgenerational +1.5°C/reproduction +0°C/development +0°C (lower right), and Transgenerational +1.5°C/reproduction +1.5°C/development +0°C (lower left). The color of the fish represents the F3 developmental condition, the smaller circle represents the conditions during parental reproduction, and the larger circle represents the developmental conditions of grandparents/parents. (d) DEGs between Transgenerational +1.5°C (top), Control +0°C/reproduction +1.5°C/development +1.5°C (lower right), and Control +0°C/reproduction +0°C/development +1.5°C (lower left)

The vast majority of DEGs were associated with developmental conditions of the F3 generation (Figure 3; Data S1), which corresponds to the most relevant environmental stimuli at the time of sampling. Specifically, the LRT showed 459 DEGs between F3 individuals that developed at Control +0°C vs. +1.5°C, regardless of the parental/grandparental conditions (Figure 3A; Figure S1). Furthermore, 327 DEGs were found when comparing +0°C vs. +1.5°C when both grandparent/parent development and F3 developmental conditions matched. Meanwhile, 355 DEGs were found when comparing +0°C vs. +1.5°C when the F3 developmental conditions matched with the parental reproduction, regardless of grandparental conditions. These results agreed with WGCNA as nine gene clusters were significantly correlated with F3 conditions (5101 genes in total; Figure S2). These gene modules showed GO enrichment for the categories: metabolism of nucleic acids, amino‐acid metabolism, immune response, oxidoreductase activity, and circadian rhythm (Data S5).

Some of the largest differences in pairwise gene expression were observed in groups that had a mismatch between parental reproductive conditions and F3 developmental conditions (Figure 3C and D). For example, the comparison between F3 individuals that developed at +0°C, from Control +0°C vs. Transgenerational +1.5°C lineages, resulted in 289 DEGs (Figure 3C). F3 individuals that developed at +0°C from the +1.5°C line showed upregulation for categories associated with stress response, cellular metabolism, increase in oxygen consumption; and downregulation for RNA translation and protein synthesis and oxygen transportation (Figure S3; Table S8, Data S6). Similar results were observed between Transgenerational +1.5°C/development +0°C vs. Transgenerational +1.5°C/reproduction +0°C/development +0°C (328 DEGs; Figure 3C; Data S7). The same pattern was observed when comparing F3 individuals that were exposed to +1.5°C for the first time during development (i.e., Control +0°C/development +1.5°C), to the Transgenerational +1.5°C lineage (187 DEGs; i.e., Transgenerational +1.5°C/reproduction +1.5°C/development +1.5°C). In this case, exposure to +1.5°C during development without previous experience showed activation for gene categories associated with cellular stress response, cell signaling, catabolism, and cellular respiration, and downregulation for biosynthesis, cardiovascular regulation, and catalysis of nucleic acids (Figure S4; Data S8). In line with these results, the comparison between F3 samples of Control +0°C vs. the Control +0°C/reproduction +0°C/development +1.5°C was an order of magnitude higher (333 DEGs; Figure S4; Data S8) than the comparison of Control +0°C vs. samples of the Transgenerational +1.5°C/reproduction +0°C/development +0°C (69 DEGs; Figure 3B; Figure S2; Table S8; Data S2). The mismatch between parental reproductive conditions and developmental conditions was significantly correlated with three gene clusters in the WGCNA (779 genes in total). These gene clusters showed GO enrichment for lipid metabolic process, oxidoreductase activity, and cofactor binding (Table S9; Data S5).

## 4 DISCUSSION

The results from this three‐generation study indicate that both previous and current generations’ thermal experience influence how *A*. *polyacanthus* responds to ocean warming. Importantly, we show that increased MO_2Max_ and AS in offspring of parents/grandparents from warmer conditions is a true transgenerational effect, regardless of the reproductive temperature of parents. Most of the DEGs associated with transgenerational exposure were related to activation of immunity and cellular stress response, which have been previously observed in the spiny chromis with increases in aerobic demand and reactive oxygen species with warming (Bernal et al., 2018; Veilleux et al., 2015). These molecular responses in concert with increased aerobic capacity suggest potential mechanisms of preconditioning to warm temperatures for future generations. In contrast, differences in other phenotypic traits (i.e., body condition and HSI) were driven by parental reproductive conditions (i.e., classic parental effect) and/or embryonic developmental exposure, highlighting how phenotypic plasticity can manifest diversely to the same environmental cues. In line with this observation, gene expression was more strongly linked to current thermal conditions of F3 fish, and whether there was a change in water temperature between parental reproduction/embryogenesis and hatching. The relatively large effect of changing temperature during early life suggests that parents precondition juveniles to their experienced/expected temperatures (i.e., predictability between parental and offspring generations), and that a shift in thermal conditions at this stage could be stressful (Pankhurst & Munday, 2011; West‐Eberhard, 2003). Thus, instances in which offspring encounter substantially different conditions than their parents during early life (e.g., marine heatwaves during the breeding season; Bernal et al., 2020; Frölicher et al., 2018) could lead to challenges for tropical marine fishes.

This study shows, for the first time, that enhanced aerobic capacity (MO_2Max_ and AS) is a transgenerational effect, with an apparent lack of influence from gametogenesis and embryonic exposure. F3 fish from grandparents/parents that developed at +1.5°C had higher MO_2Max_ regardless of the thermal conditions applied during parental reproduction, and when they developed in control conditions (i.e., no thermal experience themselves). Due to parental care in this species, previous work has been unable to disentangle the effects of reproductive and embryonic exposure from thermal developmental conditions of parents (Bernal et al., 2018; Ryu et al., 2018; Veilleux et al., 2015). These findings represent a step forward in understanding the role that transgenerational plasticity will play in response to environmental change, as for some phenotypic traits developmental exposure of previous generations is required (MO_2Max_, AS, and sex ratios; Donelson & Munday, 2015), while in other cases beneficial phenotypes can be produced without the need for previous generation's experience (MO_2Routine_, HSI, and body condition). As there was no evidence for an interaction between previous generations’ thermal history and current developmental conditions, we consider this transgenerational response to be a carry‐over effect. Transgenerational effects were also observed through the analyses of gene expression, as the pairwise comparisons of treatments with different grandparental/parental developmental temperatures showed a larger number of DEGs between these treatments when F3 individuals developed at Control compared to those that developed at +1.5°C. The development of grandparents/parents at +1.5°C showed activation of genes associated with inflammatory response and immunity, as well as GO categories associated with catabolism, metabolism of nucleic acids, oxidoreductase activity, iron binding, and signal transduction. These categories could be potentially associated with preconditioning subsequent generations to the effects of warming.

Parental reproductive temperature resulted in anticipatory parental effects (Burgess & Marshall, 2014; Engqvist & Reinhold, 2016) with an influence on MO_2Routine_, HSI, and body condition, dependent on the grandparents’/parents’ thermal history. These phenotypic traits show distinctively that there are benefits to matching thermal history, as F3 individuals from grandparents/parents developing in Control had better body condition and MO_2Routine_ when reproduction and development happened in Control conditions. Moreover, if parental reproduction occurred at +1.5°C the performance of F3 fish was improved when they developed in +1.5°C conditions, showing the benefits of matching environments between reproduction and offspring development. This suggests that these anticipatory parental effects are likely to be adaptive, which concurs with their life history as this species lacks a dispersive larval phase and parental conditions during reproduction effectively predict the offspring's environment (Burgess & Marshall, 2014; Burton & Metcalfe, 2014; Marshall & Uller, 2007). This was also observed in the Transgenerational +1.5°C line, where MO_2Routine_ was reduced and body condition was increased when offspring also developed in +1.5°C conditions. This resulted in similarities for these traits between the Control +0°C/reproduction +1.5°C and Transgenerational +1.5°C lineages, suggesting that exposure to warming during the reproductive phases of parents can result in patterns similar to transgenerational exposure for certain traits.

Considering that MO_2Routine_ and physical condition are sensitive to environmental conditions during reproduction, it was perhaps unsurprising that thermal exposure resulted in interactions between grandparent/parent developmental conditions and parental reproduction, when all offspring developed at Control +0°C. Interestingly, MO_2Routine_ of F3 offspring was affected by reproductive temperature when grandparent/parent developed at +1.5°C, while body condition was influenced by reproductive temperature when grandparent/parent developed at Control. Furthermore, when considering the effects of F3 developmental temperature on the Control line, there was evidence that higher condition (i.e., an ecologically beneficial phenotype) was observed when development and reproductive conditions matched. While the equivalent treatment in the Transgenerational +1.5°C line is absent, similar patterns are observed in the Control +0°C/reproduction +1.5°C line (i.e., matching F2 reproductive and F3 development conditions). This supports the idea that matching offspring development and parental reproductive conditions is beneficial, indicating that conditions during parental reproduction can lead to adaptive parental effects in this species.

Overall, parental reproductive conditions by themselves appear to have narrow consequences on gene expression. Disregarding F3 developmental temperature, parental reproduction at +1.5°C was associated with molecular pathways such as electron transport activity, oxidoreduction, mitochondrial activity, myosin complex, toxin metabolism, cell growth, and circadian regulation, suggesting some signatures of stress during embryogenesis. Based on the limited number of DEGs, it is possible that the relatively short length of thermal exposure during reproduction is (i.e., 6 months of reproductive exposure vs. 3.5 years over two generations prior, and 1 year of exposure following; Figure 1), not sufficient time to induce large changes on F3 juveniles. This hypothesis would be in line with theoretical perspectives that suggest reliability of cues both within the lifetime of an individual and across generations is important for induction of plasticity (Herman et al., 2014; Leimar & McNamara, 2015; Reed et al., 2010). This suggests that the combined exposure of developmental and reproductive temperatures of parents is critical for inducing future plastic change, especially for species associated with ecosystems characterized by narrow thermal variation, such as tropical species with high site fidelity.

Environmental conditions experienced in early life are known to have substantial and long‐lasting effects on later life stages (i.e., developmental plasticity; Metcalfe & Monaghan, 2001; West‐Eberhard, 2003). Our research indicates that it is not just what thermal conditions are experienced (i.e., Control or Warm), but whether a change in environment occurred during sensitive developmental stages. The analyses of gene expression highlight how mismatches between reproductive/embryonic and posthatching developmental conditions can lead to substantial effects on the F3 generation. This could be associated with the fact that many epigenetic mechanisms operate during gametogenesis and embryogenesis, which could be prominent in species with direct development, such as *A*. *polyacanthus* (Robertson, 1973). Thus, a change in thermal conditions at hatching results in the activation of molecular mechanisms associated with immunity, inflammation, cellular defense, lipid metabolism, nucleic acid replication, oxidoreduction, activity of chemokine receptors, and respiratory electron transport chain. While much of the differential gene expression is likely to indicate thermal stress, regulation of gene expression could also be considered a component of plasticity as populations of *A*. *polyacanthus* that are more heat tolerant show higher fluctuations in expression, when compared to more thermally sensitive populations (Veilleux et al., 2018). Meanwhile, individuals that were well adjusted to their conditions, thanks to matching of the F3 conditions and previous generations, showed activation of mechanisms related to oxygen transport, hemoglobin activity, structural cellular components (e.g., microtobules, centromeres), protein binding and transcription (e.g., Cdc73/Paf1 complex). It is likely that this suite of DEGs are part of the phenotypic changes observed in F3 individuals, or they could be associated with additional phenotypic traits not measured by our study.

While differences between grandparental/parental lines could lead to selection of heat‐tolerant genotypes, there was no skew in the reproductively active F1 and F2 pairs, and most of the starting great‐grandparent F0 pairs were represented in the transgenerational/reproductive treatments, suggesting selection is not the driver of the observed results (Table S9). While we found sensitivity to environmental conditions during reproduction in offspring physical condition and MO_2Routine_, this was not the case for MO_2Max_ and AS, which were transgenerationally influenced by grandparent/parental developmental conditions. Since environmental variation in nature is comprised of predictable and unpredictable components, this experiment highlights how multiple types of plastic responses can act on different traits in unison (Simons, 2014). It is how thermal plasticity of traits ultimately combines to affect fitness that is critical to understand in future research. While we found that increased aerobic capacity occurred, it remains to be determined how long the transgenerational response lasts if the thermal stimulus is no longer present (Leimar & McNamara, 2015; Nelson & Nadeau, 2010). From a climate change perspective, enhanced aerobic capacity is an ecologically relevant transgenerational effect that, accompanied by changes in gene expression, could lead to improved performance as average ocean temperatures rise. However, we know from previous research on this species that enhancement of aerobic physiology is not directly linked to improvement in reproduction, highlighting that all traits do not necessarily exhibit the same capacity for plasticity (Donelson et al., 2011, 2014, 2016). This study also highlights the benefits of complex warming exposures across generations, expanding our understanding on how populations may respond to acute warming events that are already taking place (i.e., marine heatwaves; Bernal et al., 2020). We find that there is potential for relatively rapid parental effects that occur with short‐term warming, that will likely be adaptive when offspring also experience these conditions during development. However, based on the results of this study there is the possibility that acute warming during parental reproduction could be stressful if the developmental conditions of the offspring are different. Overall, the results from this study emphasize how the experiences of both current and previous generations are important determinants of the responses to warming observed in tropical marine organisms.

## CONFLICT OF INTEREST

The authors have no conflicts of interest to disclose.

## Supporting information

Supplementary Material

Data S1‐13

## Data Availability

Phenotypic data are available at the Tropical Data Hub Research Data repository https://doi.org/10.25903/5ecc68728ec57. Raw sequences for RNA‐Seq are available on NCBI BioProject PRJNA630749 (SRA SUB7397005). The code used for the phenotypic statistical analyses and gene expression comparisons is available in GitHub (https://github.com/evofish/F3‐manuscript‐1).
