# Supplementary material for: Plasticity to ocean warming is influenced by transgenerational, reproductive, and developmental exposure in a coral reef fish
Source: Evol Appl. 2022 Jan 18;15(2):249–61. doi: 10.1111/eva.13337 (PMC8867710; doi:10.1111/eva.13337)
Supplement: Supplementary file 1 — Supplementary Material [file EVA-15-249-s002.docx]

Supplementary Materials for:

**Plasticity to ocean warming requires transgenerational exposure in a coral reef fish**

**Authors:** Moisés A. Bernal*, Timothy Ravasi, Giverny G. Rodgers, Philip L. Munday, Jennifer M. Donelson^*^

***Corresponding authors:**

Moises Bernal: [mab0205@auburn.edu](mailto:mab0205@auburn.edu); Jennifer Donelson: [Jennifer.donelson@my.jcu.edu.au](mailto:Jennifer.donelson@my.jcu.edu.au)

**This PDF file includes:**

**Figure S1.** Principal Coordinate Analysis (PCoA) of F3 thermal conditions.

**Figure S2**. Weighted Gene Correlation Network Analysis.

**Figure S3**. Gene Ontology enrichment analysis with Mann-Whitney U test of ranks (GO-MWU), between Control and Transgenerational +1.5˚C/reproduction +0˚C/development +0˚C.

**Figure S4**. Gene Ontology enrichment analysis with Mann-Whitney U test of ranks (GO-MWU), between developmental +1.5˚C and Transgenerational +1.5˚C.

**Figure S5**. Gene Ontology enrichment analysis with Mann-Whitney U test of ranks (GO-MWU), between Control and developmental +1.5˚C.

**Table S1.** Number of samples analyzed per-treatment for both phenotypic measure (oxygen consumption, weight, size, liver weight) and gene expression per treatment.

**Table S2.** Number of F3 samples analyzed by family, for both phenotypic measures and gene expression.

**Table S3.** Statistical summary of the linear model and planned comparisons for maximum oxygen consumption (MO_2Max_).

**Table S4.** Statistical summary of the linear model and planned comparisons for absolute aerobic scope (AS).

**Table S5.** Statistical summary of the linear model and planned comparisons for routine oxygen consumption (MO_2Routine_).

**Table S6.** Statistical summary of the linear model and planned comparisons for Fulton’s K condition index (FK).

**Table S7.** Statistical summary of the linear model and planned comparisons for Hepato-somatic index (HSI).

**Table S8.** Gene modules with significant *(P<*0.05) association to the experimental conditions.

**Table S9.** Gene Ontology Enrichment Analysis with the Mann-Whitney U Test for comparisons between groups that maintained their thermal conditions for three generations, and those exposed to a mismatch between parental reproduction and F3 developmental conditions.

**The Supplementary Data for this manuscript includes:**

**Data S1.** Differentially Expressed Genes detected with the Likelihood Ratio Tests.

**Data S2.** Gene Ontology terms enriched for the pairwise comparison of Control vs Developmental +1.5°C.

**Data S3.** Gene Ontology terms enriched for the pairwise comparison of Control vs Control +0°C / Reproduction +1.5°C/ Development +0°C

**Data S4.** Gene Ontology terms enriched for the pairwise comparison of Developmental +1.5°C vs Transgenerational +1.5°C

**Data S5.** Gene Ontology terms enriched for the pairwise comparison of Control +0°C vs Control +0°C/Reproduction +1.5°C/Development +1.5°C

**Data S6.** Gene Ontology terms enriched for the pairwise comparison of Transgenerational +1.5°C vs Transgenerational +1.5°C/Reproduction +0°C/Development +0°C

**Data S7.** Gene Ontology terms enriched for the pairwise comparison of Control +0°C vs Transgenerational +1.5°C/Reproduction +0°C/Development +0°C

**Data S8.** Gene Ontology terms enriched for the pairwise comparison of Transgenerational +1.5°C/Reproduction +1.5°C/Developmental +0°C vs Transgenerational +1.5°C/Reproduction +0°C/Development +0°C

**Data S9.** Gene Ontology terms enriched for the pairwise comparison of Transgenerational +1.5°C/Reproduction +1.5°C/Developmental +0°C vs Control +0°C/Reproduction +1.5°C/Development +0°C

**Data S10.** Gene Ontology terms enriched for the pairwise comparison of Developmental +1.5°C vs Control +0°C/Reproduction +1.5°C/Development +1.5°C

**Data S11.** Gene Ontology terms enriched for the pairwise comparison of Control +0°C/Reproduction +1.5°C/Development +1.5°C vs Transgenerational +1.5°C

**Data S12.** Gene Ontology terms enriched for the different modules detected with the Weighted Gene Co-expression Network Analysis

**SUPPLEMENTARY FIGURES**

Figure S1. **Principal Coordinate Analysis (PCoA) of F3 thermal conditions.** The PCoA was based on the 459 differentially expressed genes (DEGs) groups of F3 juveniles exposed Control conditions (A, LABtdA, sA, LBAt) and those exposed to +1.5˚C developmental conditions (B, LABt, ttB). The analysis excludes DEGs from grandparental/parental and reproductive temperatures. This expands on the description of Figure 1 on the main text, by presenting the distribution of the specific treatments.


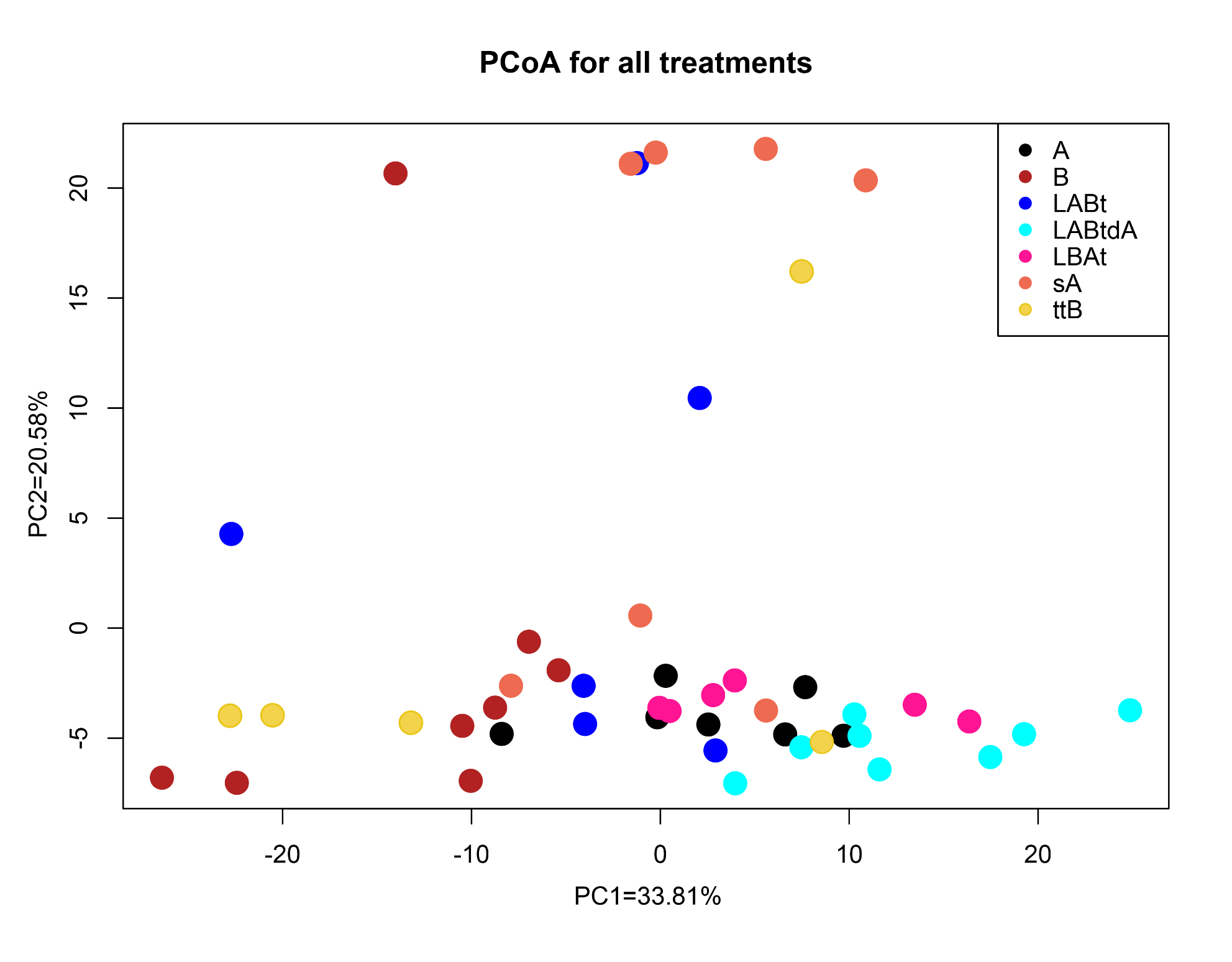


**Figure S2. Weighted Gene Correlation Network Analysis.** The different color-modules show associations of genes with similar expression. The numbers in the boxes represent the strength of the Pearson correlation between gene-modules and experimental traits: thermal conditions of the grandparents (control or warm), temperatures during parental reproduction (control or warm), developmental conditions of F3 juveniles (control or warm), whether the F3 juveniles experienced a mismatch in temperature with respect to parental reproduction (yes or no), and sex (male, female and unknown). The number below represents the corrected p-value, where values in black boxes were considered significant (*P*<0.05).


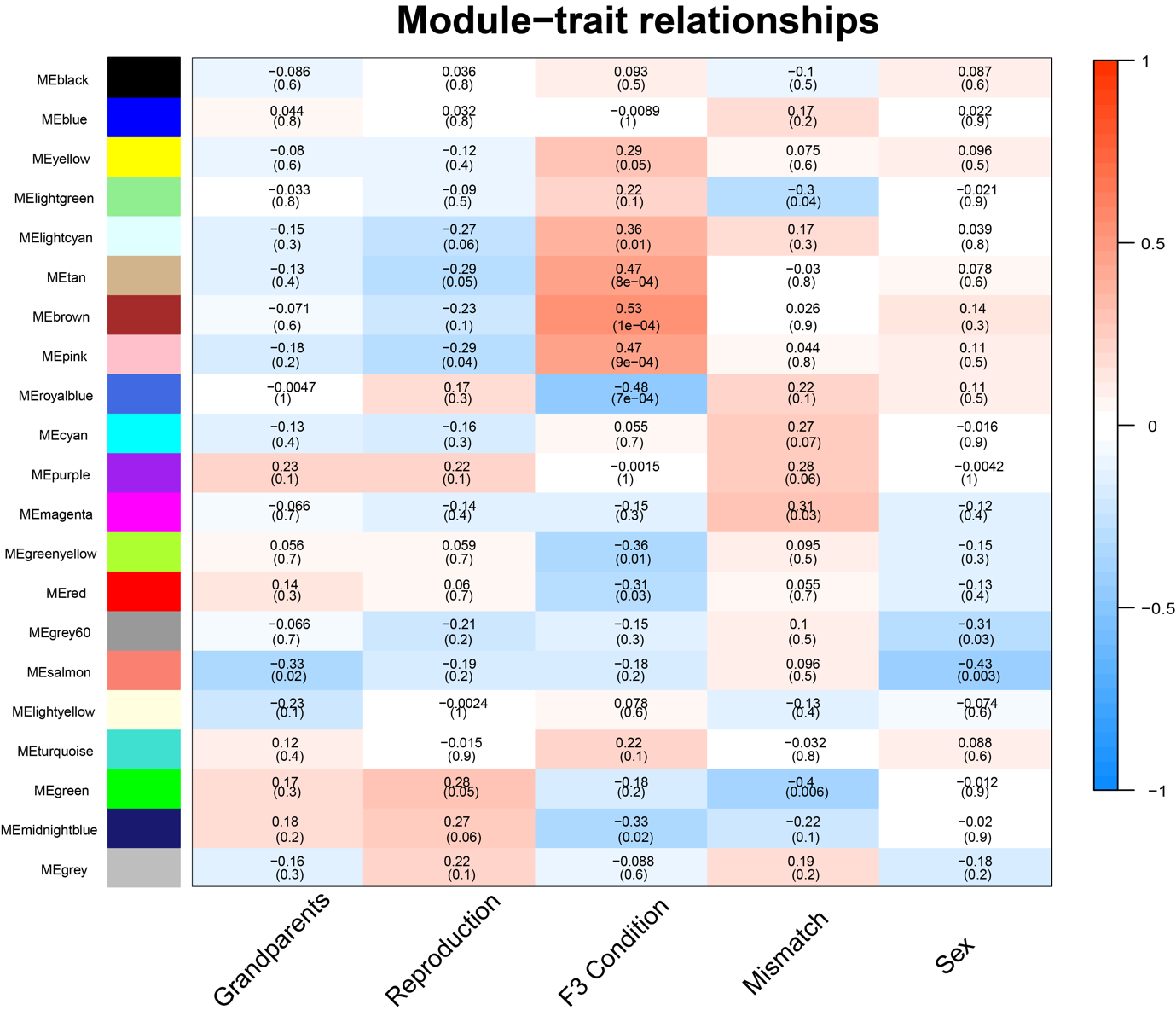


**Figure S3. Gene Ontology enrichment analysis with Mann-Whitney U test of ranks (GO-MWU), between Control and Transgenerational +1.5˚C/reproduction +0˚C/development +0˚C.** The analysis was performed for the GO subcategories (A) Biological Process (BP) and (B) Molecular Function (MF). GO terms in red denote processes downregulated in Transgenerational +1.5˚C/reproduction +0˚C/development +0˚C, while those in blue are upregulated. Font size and brightness of GO terms denotes the level of significance (large and bold=*Padj*<0.01, medium= *Padj*<0.05, small and italicized *Padj*<0.1). Numbers that precede the category name are the genes present in this particular analysis, over the total number of genes that belong to that category. Only one category was differentiated for Cellular Component, which MCM Complex (7 sequences, *P*<0.001, delta rank=1632), being activated for Transgenerational +1.5˚C/reproduction +0˚C/development +0˚C.

A

B

**Figure S4.** **Gene Ontology enrichment analysis with Mann-Whitney U test of ranks (GO-MWU), between developmental +1.5˚C and Transgenerational +1.5˚C.** The analysis was performed for the GO subcategories (A) Biological Process (BP), (B) Cellular Component (CC), and (C) Molecular Function (MF). GO terms in red denote upregulated processes in developmental +1.5˚C, while those in blue are downregulated. Font size and brightness of GO terms denotes the level of significance (large and bold= *Padj*<0.01, medium= *Padj*<0.05, small and italicized *Padj*<0.1). Numbers that precede the category name are the genes present in this particular analysis, over the total number of genes that belong to that category.

A C

B

**Figure S5. Gene Ontology enrichment analysis with Mann-Whitney U test of ranks (GO-MWU), between Control and developmental +1.5˚C.** The analysis was performed for the GO subcategories (A) Biological Process (BP), (B) Cellular Component (CC), and (C) Molecular Function (MF). GO terms in red denote downregulated processes in developmental +1.5˚C, while those in blue are upregulated. Font size and brightness of GO terms denotes the level of significance (large and bold= *Padj*<0.01, medium= *Padj*<0.05, small and italicized *Padj*<0.1). Numbers that precede the category name are the genes present in this particular analysis, over the total number of genes that belong to that category.

C

A

B

**SUPPLEMENTARY TABLES**

**Table S1.** Number of samples analyzed per-treatment separated by sex, for both phenotypic measure (weight, size, liver weight and body condition) and gene expression. Sex was not evaluated for individuals measured in respirometry assays, thus it has been omitted from this table. Sample sizes for respirometry are available on Table 1 of the main text.

| Treatment | Phenotype: HSI and body condition | | | Gene Expression | | |
| --- | --- | --- | --- | --- | --- | --- |
|  | Male | Female | Und | Male | Female | Und |
| Control +0°C | 2 | 5 | 1 | 2 | 5 | - |
| Developmental +1.5°C | 5 | 3 | 1 | 5 | 3 | - |
| Control +0°C /reproduction +1.5°C/development +0°C | 6 | 3 | - | 3 | 5 | - |
| Control +0°C /reproduction +1.5°C/development +1.5°C | 3 | 3 | - | 3 | 3 | - |
| Transgenerational +1.5°C/reproduction +0°C/ development +0°C | 6 | 5 | - | 3 | 2 | 1 |
| Transgenerational +1.5°C/reproduction +1.5°C/developmental +0°C | 3 | 2 | 1 | 3 | 4 | - |
| Transgenerational +1.5°C | 3 | 3 | - | 3 | 2 | - |

**Table S2.** Number of F3 samples analyzed by family, for both phenotypic measures and gene expression. Each row indicates a replicate F2 pair and the composition of that pair linking back to the eight F0 wild pairs (3, 6, 14, 37, 41, 48, 68 and 69). The column “Treatment” represents the transgenerational conditions applied to the specific lineage. The numbers in italics represent the temperatures applied in the F3 generation.

| Treatment | F2 Male | F2 Female | F2 male | | F2 female | | Aerobic metabolism | | HSI and body condition | | Gene expression | |
| --- | --- | --- | --- | --- | --- | --- | --- | --- | --- | --- | --- | --- |
|  |  |  | F1 father | F1 mother | F1 father | F1 mother |  |  |  |  |  |  |
|  |  |  |  |  |  |  | *+0°C* | *+1.5°C* | *+0°C* | *+1.5°C* | *+0°C* | *+1.5°C* |
| Control +0°C | 31/127 | 79/107 | 68 | 48 | 69 | 14 | 5 | 7 | 1 | 7 | 1 | 6 |
|  | 32/118 | 79/107 | 37 | 6 | 69 | 14 | 6 | 2 | 3 | 0 | 3 | 0 |
|  | 31/127 | 108/35 | 68 | 48 | 69 | 41 | 4 | 2 | 2 | 1 | 1 | 1 |
|  | 77/102 | 86/104 | 6 | 14 | 6 | 41 | 6 | 1 | 2 | 1 | 2 | 1 |
| Control +0°C /reproduction +1.5°C | 79/107 | 76/106 | 69 | 14 | 48 | 6 | 2 | 5 | 2 | 1 | 2 | 1 |
|  | 108/35 | 43/64 | 69 | 41 | 48 | 41 | 2 | 3 | 2 | 1 | 1 | 1 |
|  | 86/104 | 31/127 | 6 | 41 | 68 | 48 | 1 | 8 | 1 | 1 | 1 | 1 |
|  | 31/127 | 79/107 | 68 | 48 | 69 | 14 | 2 | 2 | 2 | 1 | 2 | 1 |
|  | 77/102 | 86/104 | 6 | 14 | 6 | 41 | 2 | 7 | 2 | 2 | 2 | 2 |
| Transgenerational +1.5°C/ reproduction +0°C | 42/60 | 17/16 | 48 | 69 | 41 | 3 | 2 | NA | 0 | NA | 0 | NA |
|  | 90/91 | 42/60 | 41 | 3 | 48 | 69 | 2 | NA | 3 | NA | 3 | NA |
|  | 17/16 | 6/40 | 41 | 3 | 48 | 37 | 3 | NA | 1 | NA | 1 | NA |
|  | 90/91 | 42/60 | 41 | 3 | 48 | 69 | 7 | NA | 1 | NA | 1 | NA |
|  | 6/40 | 11/12 | 48 | 37 | 41 | 48 | 2 | NA | 1 | NA | 1 | NA |
| Transgenerational +1.5°C | 90/91 | 17/16 | 41 | 3 | 41 | 3 | 5 | 5 | 2 | 1 | 2 | 1 |
|  | 14/15 | 61/82 | 68 | 41 | 14 | 68 | 5 | 2 | 2 | 1 | 2 | 1 |
|  | 14/15 | 6/40 | 68 | 41 | 48 | 37 | 5 | 9 | 5 | 4 | 1 | 3 |
|  | 17/16 | 6/40 | 41 | 3 | 48 | 37 | 5 | 1 | 2 | 1 | 2 | 1 |

**Table S3:** Statistical summary of the linear mixed effects model and planned comparisons for maximum oxygen consumption (MO_2Max_).

|  | Estimate | Std. error | t value | Significance |
| --- | --- | --- | --- | --- |
| Full model |  |  |  |  |
| log weight | -0.1358 | 0.0836 | -1.625 | **-** |
| Intercept: +0°C | 2.8534 | 0.0995 | 28.669 | **-** |
| Grandparent/parent development +1.5°C | 0.0672 | 0.0580 | 1.158 | - |
| Parent reproduction +1.5°C | -0.0200 | 0.0614 | -0.327 | - |
| Development 1.5°C | -0.0405 | 0.0354 | -1.143 | **-** |
| Grandparent/parent development +1.5°C: parent reproduction +1.5°C | 0.0841 | 0.0848 | 1.625 | **-** |
| Grandparent/parent development +1.5°C: development +1.5°C | -0.1062 | 0.0513 | -2.069 | - |
| Parent reproduction +1.5°C: development +1.5°C | 0.0771 | 0.0519 | 1.485 | - |
| *Planned comparisons: F3 +0°C Control only* | |  |  |  |
| Grandparent/parent development +0 vs +1.5°C | -0.1093 | 0.0434 | -2.520 | **0.020** |
| Parent reproduction +0 vs +1.5°C | -0.0220 | 0.0424 | -0.520 | 0.609 |
| Interaction between Grandparent/parent development and parent reproduction | 0.0421 | 0.0425 | 0.990 | 0.334 |
| *Planned comparisons: excluding Transgenerational +1.5°C, reproduction +0°C line* | | | |  |
| Development +0 vs +1.5°C | 0.0245 | 0.0210 | 1.168 | 0.246 |
| Interaction between Control +0°C vs Control +0°C reproducing at +1.5°C lineage depending on developmental temperature | 0.0385 | 0.0261 | 1.478 | 0.143 |
| Interaction between Control +0°C reproducing at +1.5°C vs Transgenerational +1.5°C lineage depending on developmental temperature | -0.0531 | 0.0258 | -2.059 | **0.042** |

**Table S4:** Statistical summary of the linear mixed effects model and planned comparisons for absolute aerobic scope (AS).

|  | Estimate | Std. error | t value | Significance |
| --- | --- | --- | --- | --- |
| Full model |  |  |  |  |
| log weight | -0.1045 | 0.1372 | -0.762 | - |
| Intercept: +0°C | 2.5882 | 0.1610 | 16.072 | - |
| Grandparent/parent development +1.5°C | 0.1316 | 0.0904 | 1.456 | - |
| Parent reproduction +1.5°C | -0.0197 | 0.0962 | -0.205 | - |
| Development 1.5°C | -0.1160 | 0.0590 | -1.968 | **-** |
| Grandparent/parent development +1.5°C: parent reproduction +1.5°C | 0.0484 | 0.1326 | 0.365 | **-** |
| Grandparent/parent development +1.5°C: development +1.5°C | -0.1316 | 0.0837 | -1.573 | - |
| Parent reproduction +1.5°C: development +1.5°C | 0.1785 | 0.0862 | 2.071 | - |
| *Planned comparisons: F3 +0°C Control only* | |  |  |  |
| Grandparent/parent development +0 vs +1.5°C | -0.1558 | 0.0678 | -2.299 | **0.031** |
| Parent reproduction +0 vs +1.5°C | -0.0045 | 0.0661 | -0.067 | 0.947 |
| Interaction between Grandparent/parent development and parent reproduction | 0.0242 | 0.0665 | 0.364 | 0.720 |
| *Planned comparisons: excluding Transgenerational +1.5°C, reproduction +0°C line* | | | |  |
| Development +0 vs +1.5°C | 0.0409 | 0.0344 | 1.190 | 0.237 |
| Interaction between Control +0°C vs Control +0°C reproducing at +1.5°C lineage depending on developmental temperature | 0.0892 | 0.0434 | 2.058 | **0.042** |
| Interaction between Control +0°C reproducing at +1.5°C vs Transgenerational +1.5°C lineage depending on developmental temperature | -0.0658 | 0.0421 | -1.564 | 0.121 |

**Table S5.** Statistical summary of the linear mixed effects model and planned comparisons for routine oxygen consumption (MO_2Routine_).

|  | Estimate | Std. error | t value | Significance |
| --- | --- | --- | --- | --- |
| Full model |  |  |  |  |
| log weight | -0.1612 | 0.0551 | -2.926 | **-** |
| Intercept: +0°C | 2.4624 | 0.0621 | 39.643 | **-** |
| Grandparent/parent development +1.5°C | -0.0494 | 0.0267 | -1.849 | - |
| Parent reproduction +1.5°C | 0.0121 | 0.0304 | 0.397 | - |
| Development 1.5°C | 0.0579 | 0.0267 | 2.172 | **-** |
| Grandparent/parent development +1.5°C: parent reproduction +1.5°C | 0.1155 | 0.0407 | 2.838 | **-** |
| Grandparent/parent development +1.5°C: development +1.5°C | -0.0450 | 0.0366 | -1.227 | - |
| Parent reproduction +1.5°C: development +1.5°C | -0.0724 | 0.0381 | -1.903 | - |
| *Planned comparisons: F3 +0°C Control only* |  |  |  |  |
| Grandparent/parent development +0 vs +1.5°C | -0.0083 | 0.0210 | -0.396 | 0.695 |
| Parent reproduction +0 vs +1.5°C | -0.0698 | 0.0206 | -3.384 | **0.002** |
| Interaction between Grandparent/parent development and parent reproduction | 0.0577 | 0.0205 | 2.816 | **0.008** |
| *Planned comparisons: excluding Transgenerational +1.5°C, reproduction +0°C line* | | | |  |
| Development +0 vs +1.5°C | 0.0053 | 0.0156 | 0.340 | 0.734 |
| Interaction between Control +0°C vs Control +0°C reproducing at +1.5°C lineage depending on developmental temperature | -0.0362 | 0.0195 | -1.858 | 0.066 |
| Interaction between Control +0°C reproducing at +1.5°C vs Transgenerational +1.5°C lineage depending on developmental temperature | -0.0225 | 0.0187 | -1.203 | 0.232 |

**Table S6:** Statistical summary of the linear mixed effects model and planned comparisons for physical condition (weight for a given standard length).

|  | Estimate | Std. error | t value | Significance |
| --- | --- | --- | --- | --- |
| Full model |  |  |  |  |
| Standard length | 0.5314 | 0.0285 | 18.666 | - |
| Intercept: +0°C | -20.817 | 1.925 | -10.816 | - |
| Grandparent/parent development +1.5°C | -1.5501 | 0.6912 | -2.243 | - |
| Parent reproduction +1.5°C | -1.2605 | 0.6271 | -2.010 | - |
| Development 1.5°C | -1.9947 | 0.5221 | -3.820 | **-** |
| Grandparent/parent development +1.5°C: parent reproduction +1.5°C | 2.2434 | 0.9309 | 2.410 | **-** |
| Grandparent/parent development +1.5°C: development +1.5°C | -1.1689 | 0.6287 | -1.859 | - |
| Parent reproduction +1.5°C: development +1.5°C | 2.832 | 0.6897 | 4.106 | - |
| *Planned comparisons: F3 +0°C Control only* | | | | |
| Grandparent/parent development +0 vs +1.5°C | 0.428 | 0.490 | 0.875 | 0.393 |
| Parent reproduction +0 vs +1.5°C | 0139 | 0.463 | 0.300 | 0.768 |
| Interaction between Grandparent/parent development and parent reproduction | 1.122 | 0.468 | 2.397 | **0.028** |
| *Planned comparisons: excluding Transgenerational +1.5°C, reproduction +0°C line* | | | | |
| Development +0 vs +1.5°C | 0.497 | 0.285 | 1.740 | 0.090 |
| Interaction between Control +0°C vs Control +0°C reproducing at +1.5°C lineage depending on developmental temperature | 1.416 | 0.354 | 3.995 | **<0.001** |
| Interaction between Control +0°C reproducing at +1.5°C vs Transgenerational +1.5°C lineage depending on developmental temperature | -0.584 | 0.315 | -1.855 | 0.072 |

**Table S7:** Statistical summary of the linear mixed effects model and planned comparisons for Hepato-somatic index (HSI).

|  | Estimate | Std. error | t value | Significance |
| --- | --- | --- | --- | --- |
| Full model |  |  |  |  |
| Intercept: +0°C | 0.01446 | 0.0015 | 9.525 | - |
| Grandparent/parent development +1.5°C | -0.0020 | 0.0022 | -0.903 | - |
| Parent reproduction +1.5°C | -0.0038 | 0.0021 | -1.842 | - |
| Development 1.5°C | -0.0039 | 0.0015 | -2.707 | **-** |
| Grandparent/parent development +1.5°C: parent reproduction +1.5°C | 0.0039 | 0.0030 | 1.280 | **-** |
| Grandparent/parent development +1.5°C: development +1.5°C | 0.0002 | 0.0017 | 0.110 | - |
| Parent reproduction +1.5°C: development +1.5°C | 0.0076 | 0.0019 | 3.989 | - |
| *Planned comparisons: F3 +0°C Control only* | | | | |
| Grandparent/parent development +0 vs +1.5°C | 0.00009 | 0.0015 | 0.062 | 0.952 |
| Parent reproduction +0 vs +1.5°C | 0.00187 | 0.0015 | 1.239 | 0.234 |
| Interaction between Grandparent/parent development and parent reproduction | 0.00193 | 0.0015 | 1.277 | 0.220 |
| *Planned comparisons: excluding Transgenerational +1.5°C, reproduction +0°C line* | | | | |
| Development +0 vs +1.5°C | -0.00117 | 0.0007 | -1.563 | 0.126 |
| Interaction between Control +0°C vs Control +0°C reproducing at +1.5°C lineage depending on developmental temperature | 0.00378 | 0.0010 | 3.927 | **0.003** |
| Interaction between Control +0°C reproducing at +1.5°C vs Transgenerational +1.5°C lineage depending on developmental temperature | 0.00009 | 0.0008 | 0.110 | 0.913 |

**Table S8.** Gene Ontology Enrichment Analysis with the Mann-Whitney U Test for comparisons between groups that maintained their thermal conditions for three generations, and those exposed to a mismatch between parental reproduction and F3 developmental conditions.

|  | Comparison | Biological  Process | Cellular  Component | Molecular  Function |
| --- | --- | --- | --- | --- |
| Control +0°C | Ctrl 0°C/repro +0°C/  dev +1.5°C | 35 | 21 | 38 |
|  | Tran +1.5°C/repro +1.5°C/  dev +0°C | 26 | 20 | 41 |
|  | Tran +1.5°C/repro +0°C/  dev +0°C | 6 | 1 | 7 |
| Transgenerational +1.5C | Ctrl 0°C/repro +0°C/  dev +1.5°C | 21 | 13 | 22 |
|  | Ctrl 0°C/repro +0°C/  dev +1.5°C | 84 | 17 | 29 |

**Table S9.** Gene modules with significant *(P<*0.05) association to the experimental conditions. The number of genes on each module and the number of GO-terms with significant enrichment for each of the ontology domains: Biological Process (BP), Cellular Component (CC) and Molecular Function (MF).

| Modules | Number of Genes  per Category | Enriched GO-terms | | |
| --- | --- | --- | --- | --- |
|  |  | **BP** | **CC** | **MF** |
| Royal-blue | 42 | 7 | 0 | 0 |
| Light-green | 83 | 1 | 3 | 3 |
| Grey-60 | 97 | 5 | 1 | 0 |
| Light-cyan | 102 | 6 | 2 | 4 |
| Midnight-blue | 126 | 0 | 0 | 0 |
| Salmon | 168 | 13 | 0 | 13 |
| Tan | 210 | 0 | 1 | 0 |
| Green-yellow | 211 | 1 | 1 | 5 |
| Magenta | 264 | 3 | 0 | 11 |
| Pink | 266 | 9 | 1 | 7 |
| Red | 340 | 1 | 1 | 7 |
| Green | 432 | 0 | 0 | 4 |
| Yellow | 468 | 0 | 1 | 7 |
| Brown | 917 | 0 | 1 | 1 |
